# Supplementary material for: Restructuring highly electron-deficient metal-metal oxides for boosting stability in acidic oxygen evolution reaction
Source: Nat Commun. 2021 Sep 28;12:5676. doi: 10.1038/s41467-021-26025-0 (PMC8479065; doi:10.1038/s41467-021-26025-0)
Supplement: Supplementary file 1 — Restructuring highly electron-deficient metal-metal oxides for boosting stability in acidic oxygen evolution reaction [file 41467_2021_26025_MOESM1_ESM.pdf]

Supporting information

Restructuring highly electron-deficient metal-metal oxides for boosting stability in acidic oxygen evolution reaction

Xinghui Liu[1,2], Shibo Xi[3], Hyunwoo Kim[4], Ashwani Kumar[1,2], Jinsun Lee[1,2], Jian Wang[5], Ngoc Quang Tran[1], Taehun Yang[1,2], Xiaodong Shao,[1,2] Mengfang Liang[1,2], Min Gyu Kim[6], Hyoyoung Lee[1,2,7*]

[1]Center for Integrated Nanostructure Physics (CINAP), Institute of Basic Science (IBS), 2066 Seoburo, Jangan-Gu, Suwon 16419, Republic of Korea.

[2]Department of Chemistry, Sungkyunkwan University (SKKU), 2066 Seoburo, Jangan-Gu, Suwon 16419, Republic of Korea.

[3]Institute of Chemical and Engineering Sciences, A∗STAR, 1 Pesek Road, Jurong Island, 627833, Singapore.

[4]Department of Energy Science, Sungkyunkwan University (SKKU), 2066 Seoburo, Jangan-Gu, Suwon 16419, Republic of Korea.

[5]Department of Chemistry, College of Science, Seoul National University, Seoul, 08826, Republic of Korea.

[6]Beamline Research Division, Pohang Accelerator Laboratory (PAL), Pohang University of Science and Technology, Pohang 37673, Republic of Korea.

[7]Department of Biophysics, Sungkyunkwan University (SKKU), 2066 Seoburo, Jangan-Gu, Suwon 16419, Republic of Korea.

[*]Corresponding author. Email: hyoyoung@skku.edu (H.L.)

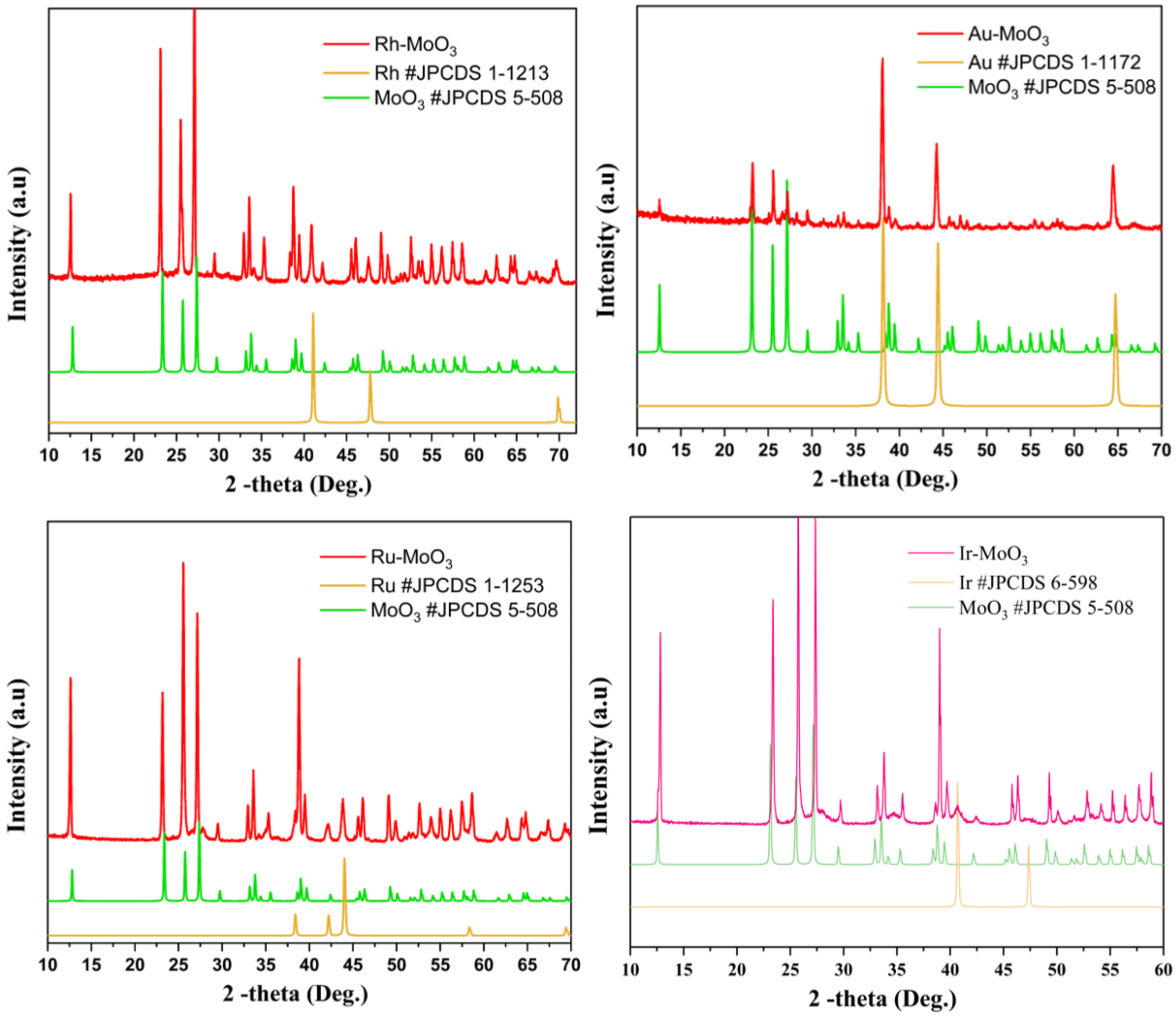

Figure S1. XRD pattern of the as-prepared metal-semiconductor nanocomposite.

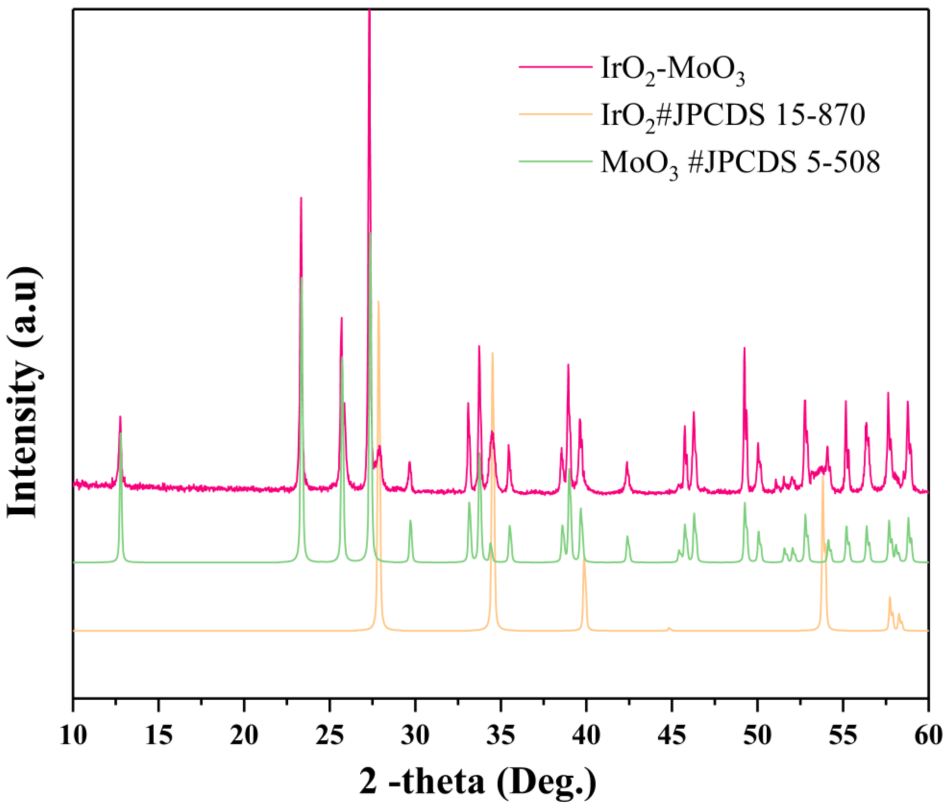

Figure S2. XRD pattern of IrO_2-MoO_3.

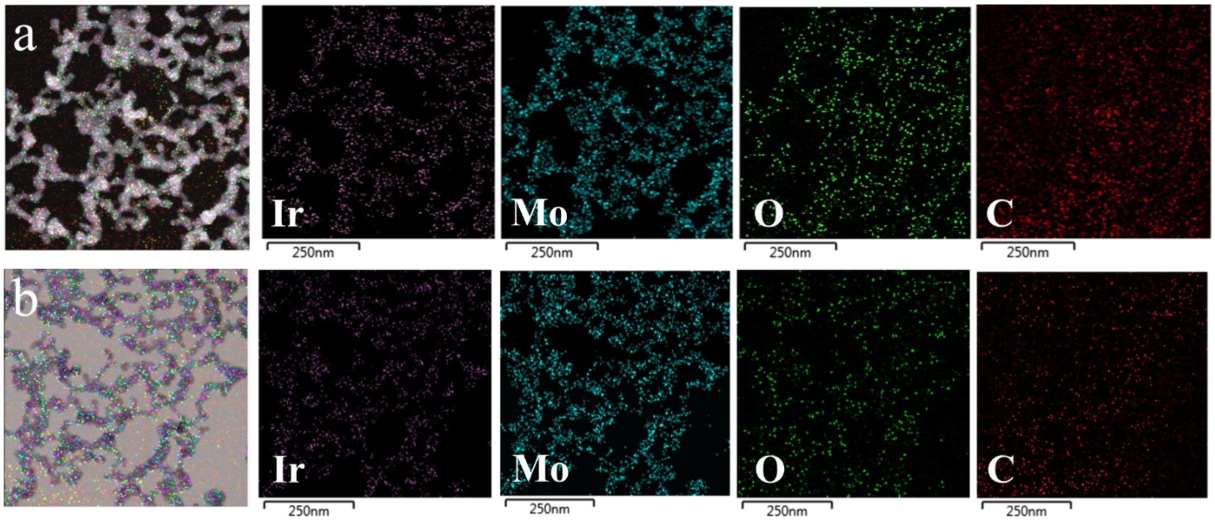

Figure S3, Structural characterization and elemental maps (Ir, Mo, O, C) of IMO. a, the high-angle annular dark-field scanning transmission electron microscopy energy-dispersive X-ray spectroscopy (HAADF-STEM-EDS). **b,** the annular bright-field scanning transmission electron microscopy energy-dispersive X-ray spectroscopy (ABF-STEM-EDS) elemental mapping images.

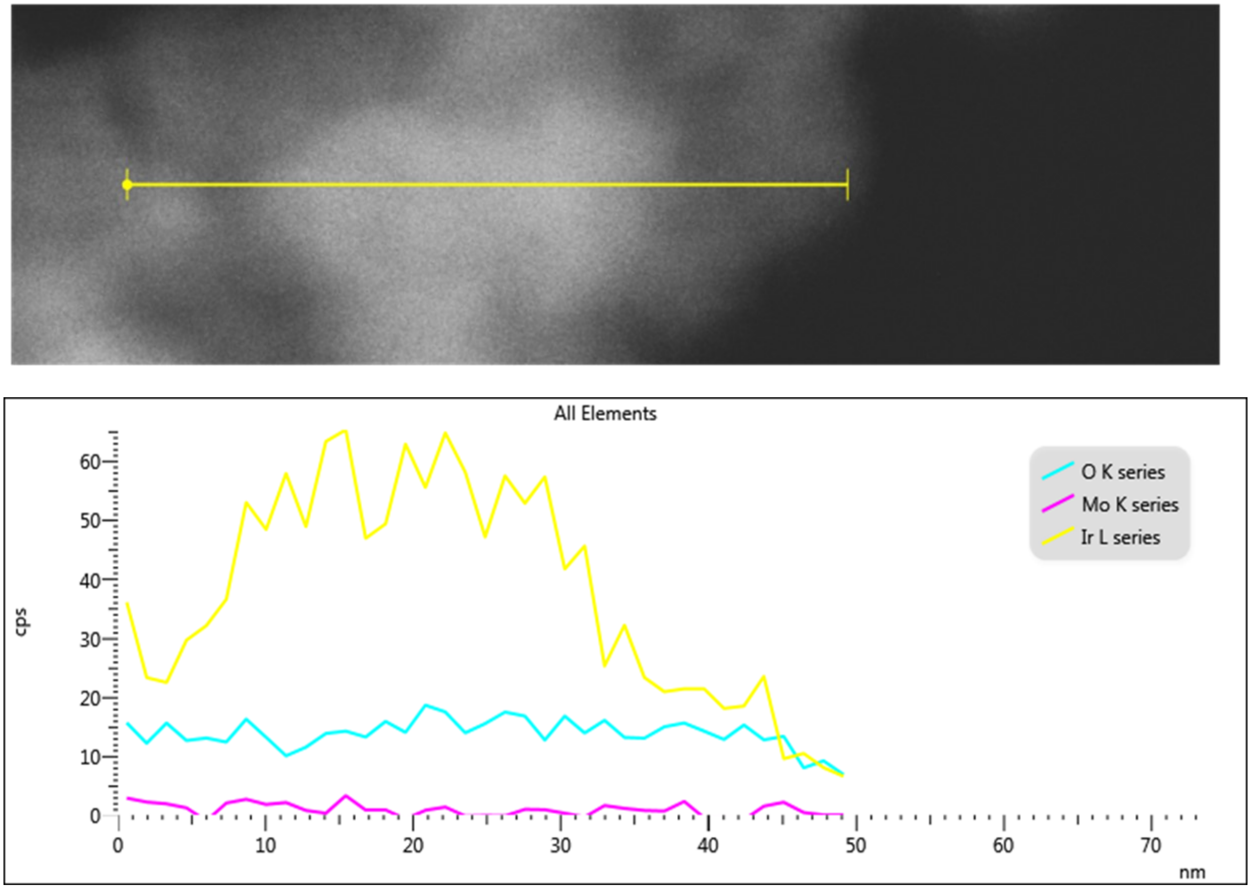

Figure S4. Line EDS mapping of IMO of Figures 2d and 2e.

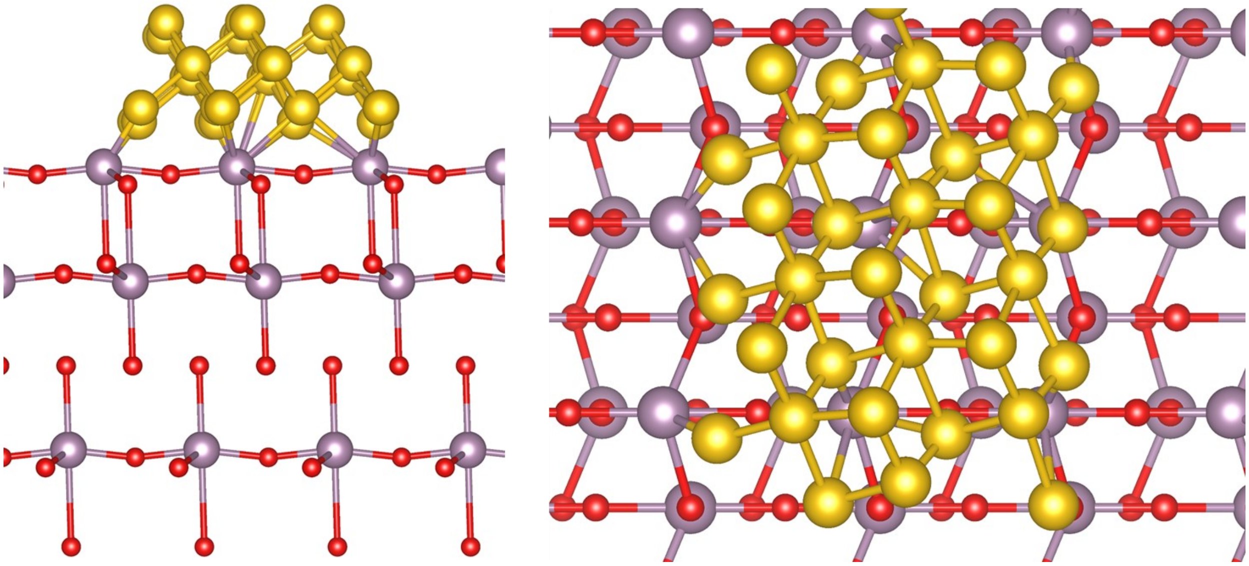

Figure S5. The side view of (left) and top view (right) of the structural model. Color code: Mo (purple), O (red), Ir (yellow).

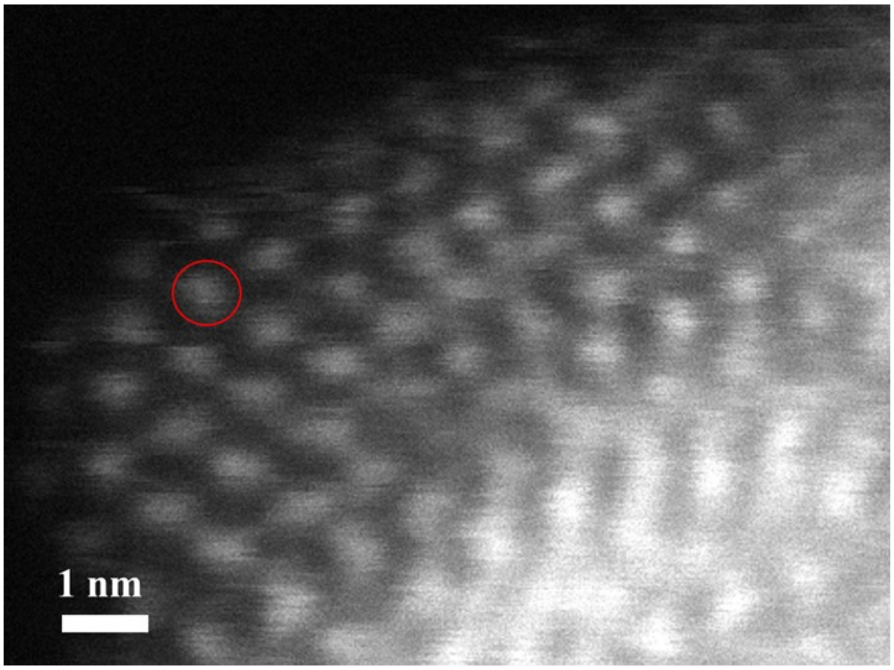

Figure S6. HAADF-STEM image of Ir metal in IMO. The bright points are Ir atoms.

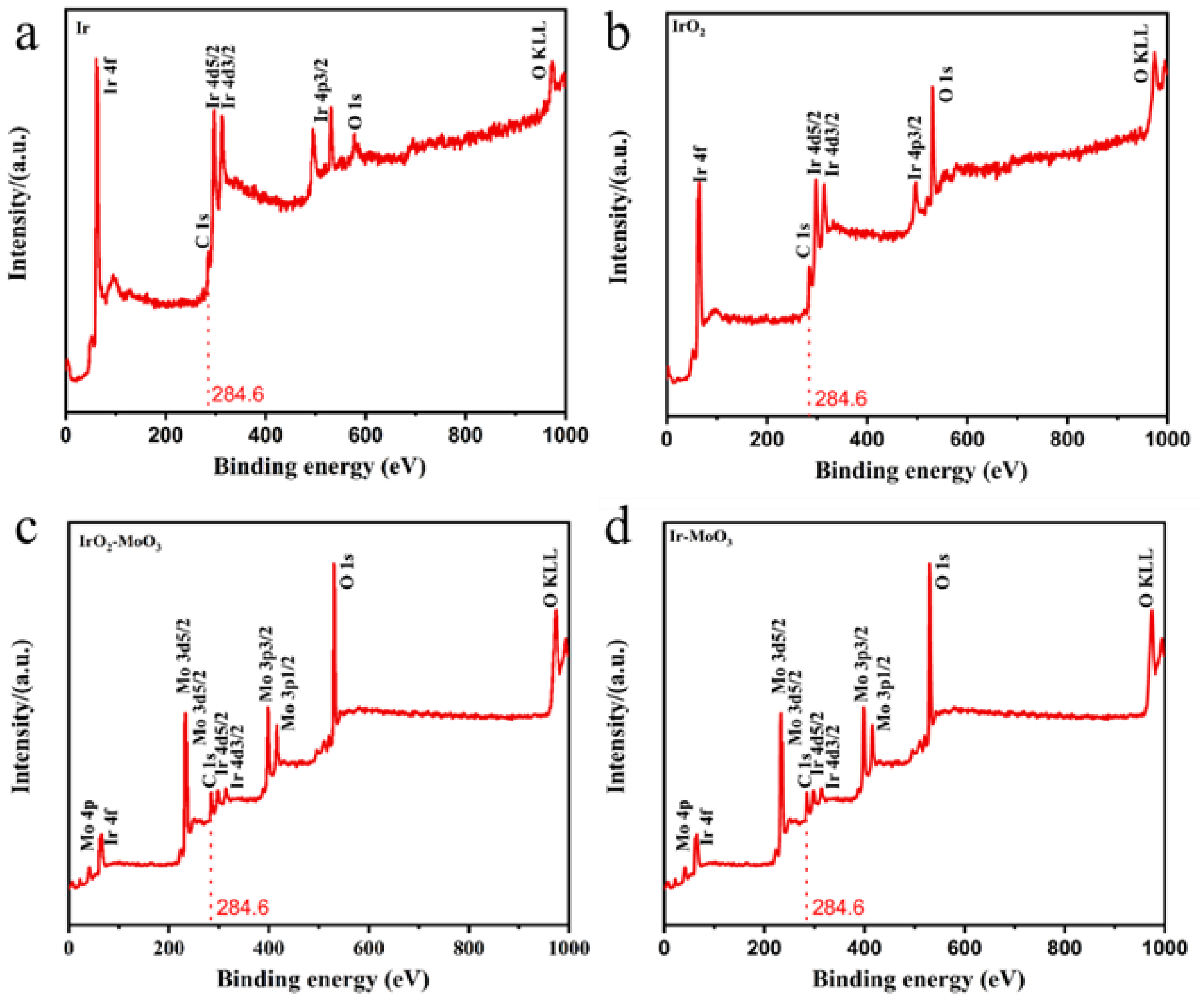

Figure S7. The survey spectrum with energy calibration using C 1s at 284.6 eV. a, Commercial Ir. **b,** Commercial IrO_2. **c,** IrO_2-MoO_3. **d,** Ir-MoO_3.

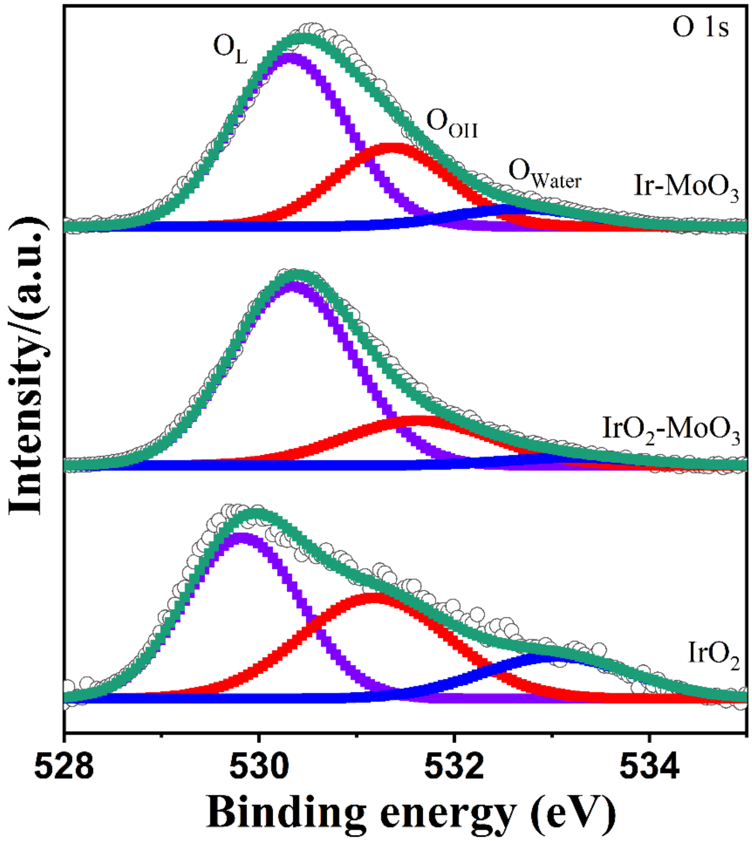

Figure S8. HR-XPS of O 1s for commercial IrO_2, IOMO, and IMO. Where O_L is lattice oxygen, O_{OH} is adsorbed hydroxide, and O_{Water} is adsorbed water.

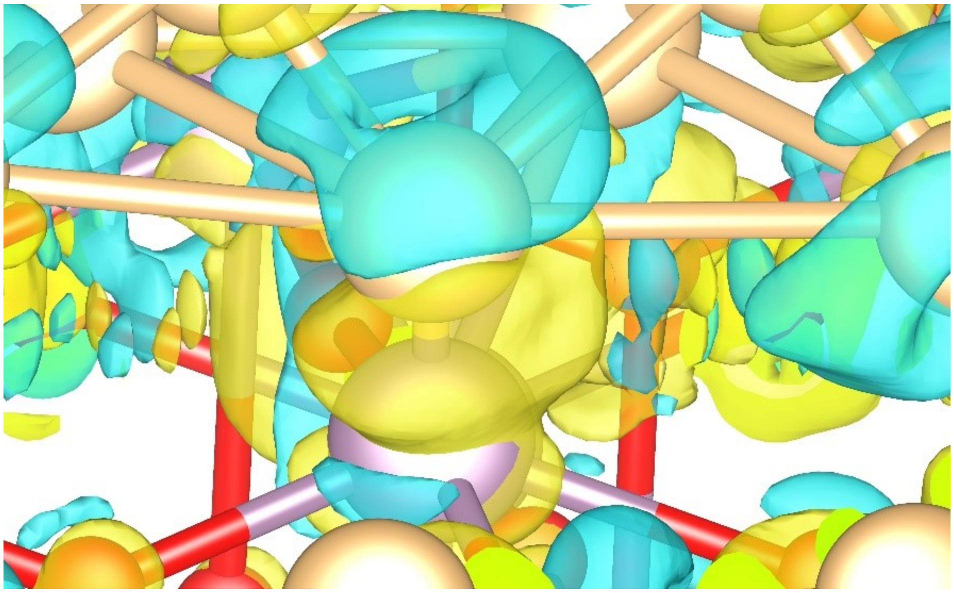
.

Figure S9. The other enlarger charge density difference images of IMO. The yellow and cyan regions represent electron accumulation and depletion. The red, gold, and violet balls represent the O, Ir, and Mo atoms, respectively. The isosurface value is 0.015e/bohr^3.

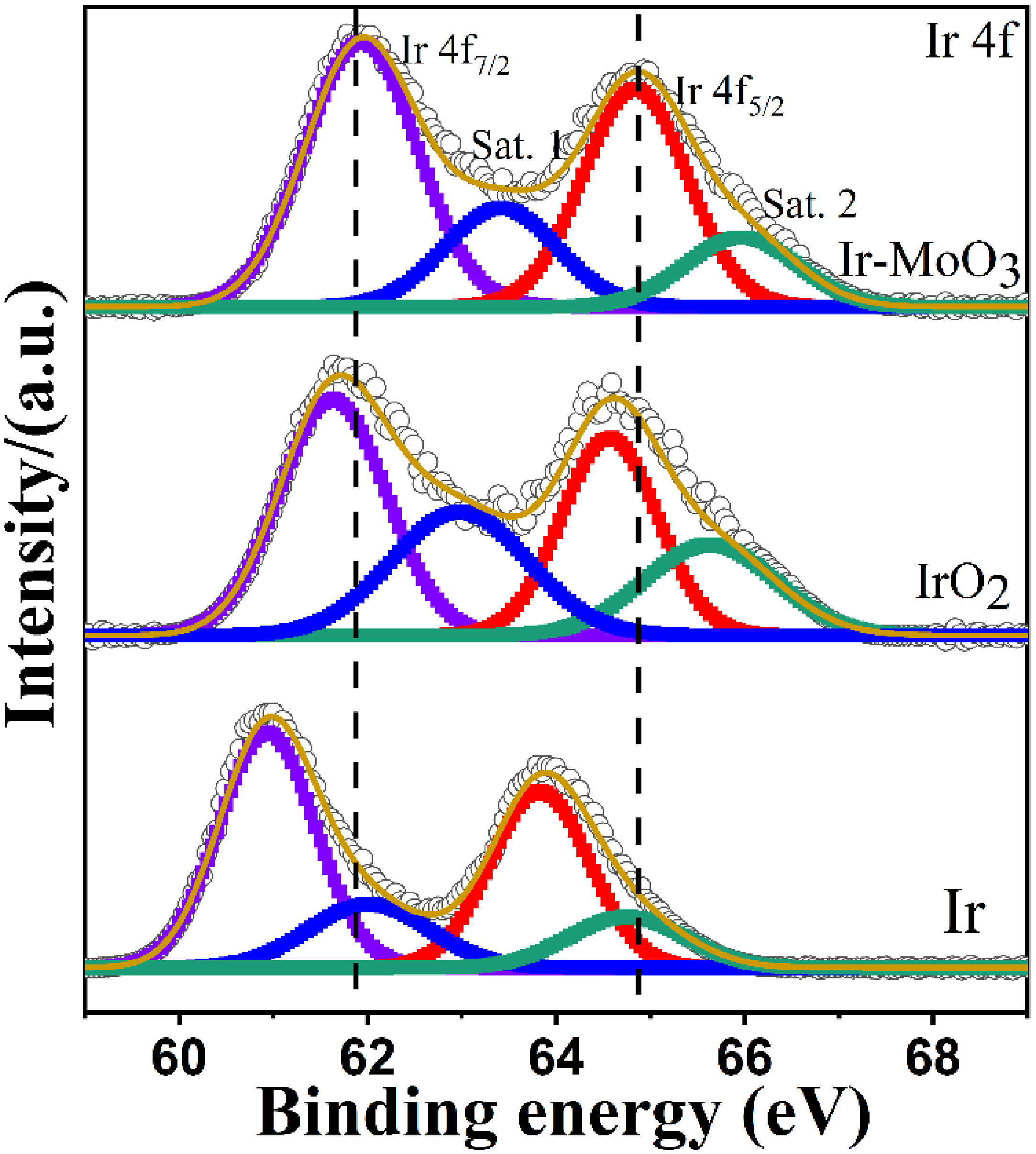

Figure S10. HR-XPS of Ir 4f for commercial Ir, IrO_2, and IMO.

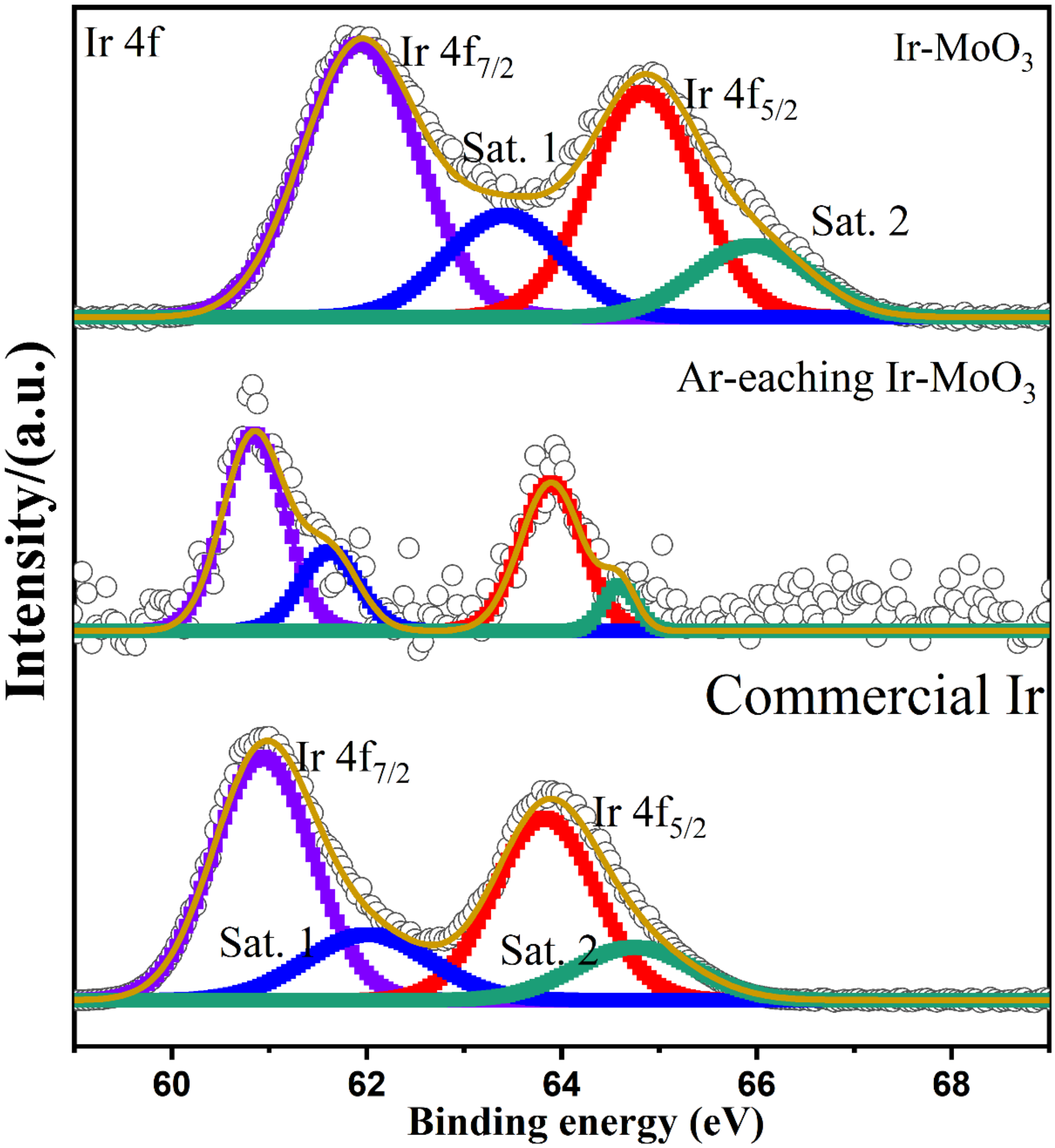

Figure S11 HR-XPS of Ir 4f for the Commercial Ir, IMO, and argon (Ar) etching IMO.

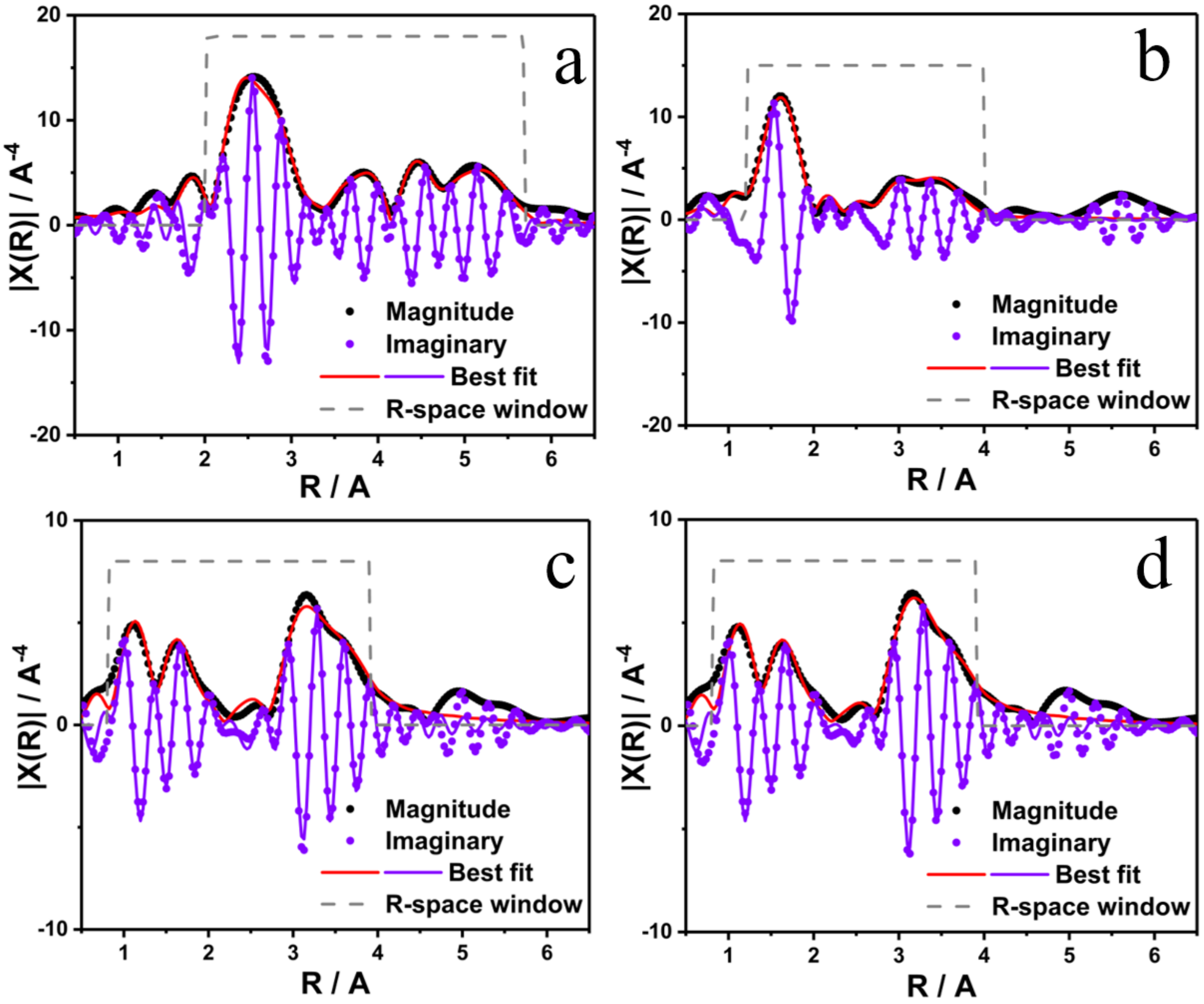

Figure S12. a, EXAFS fitting curve for Ir in IMO; **b,** EXAFS fitting curve for Ir in IOMO; **c,** EXAFS fitting curve for Mo in IMO; **d,** EXAFS fitting curve for Mo in IOMO.

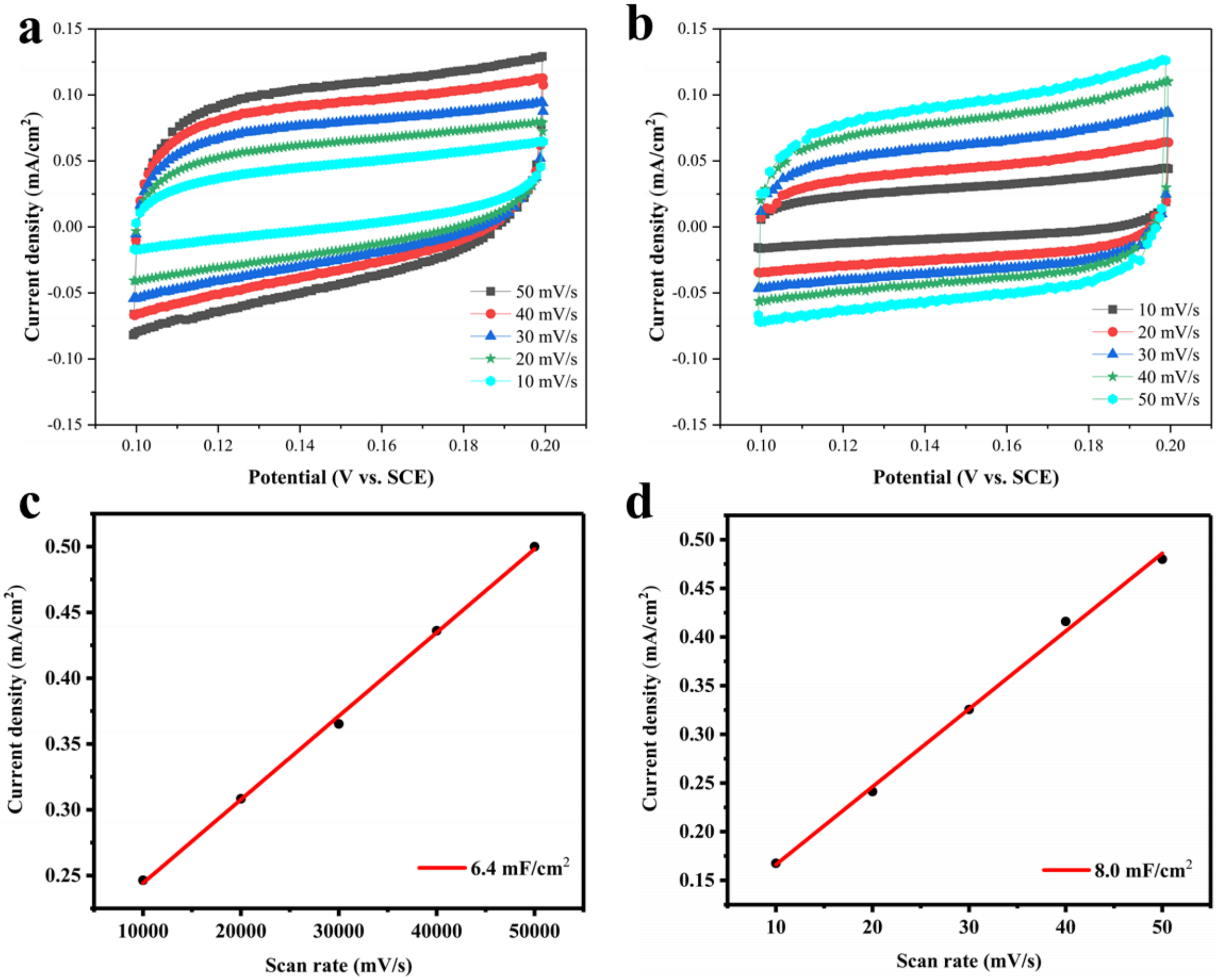

Figure S13. a, Cyclic voltammograms of IOMO acquired at the scan rates of 10 mV to 50 mV per second. **b,** Cyclic voltammograms of IMO acquired at the scan rates of 10 mV to 50 mV per second. **c,** The corresponding plot for estimating C_{DL} of IOMO **d,** The corresponding plot for estimating C_{DL} of IMO.

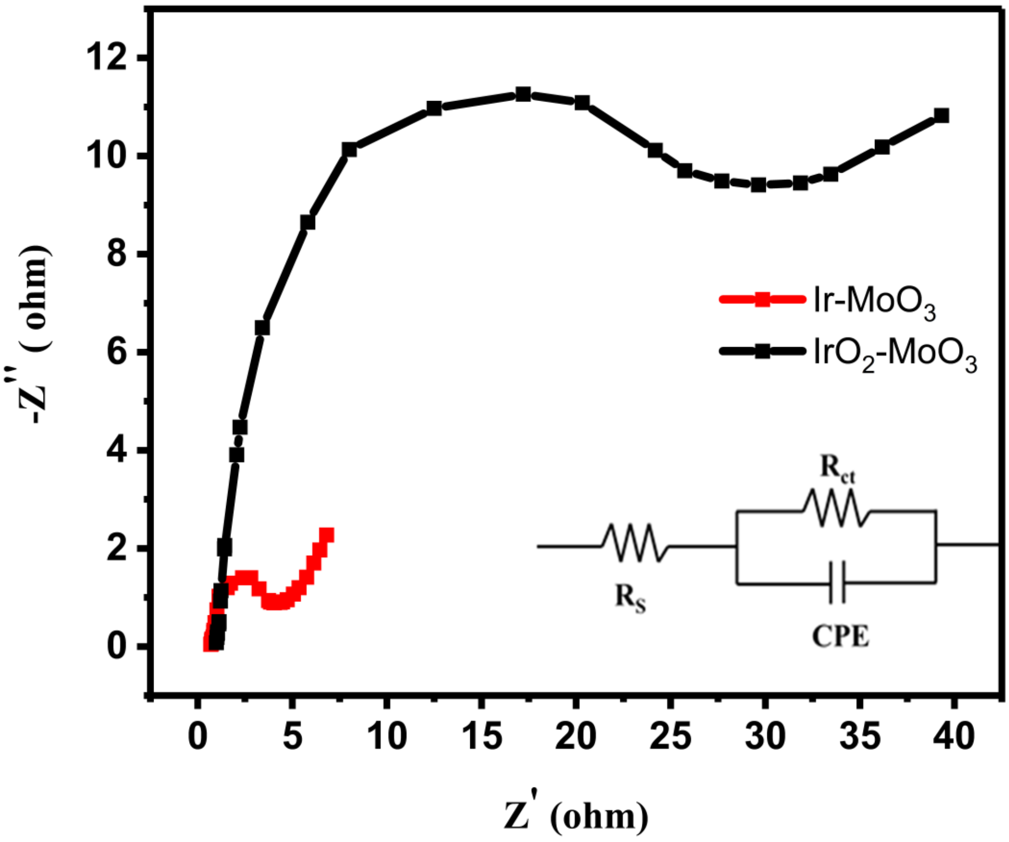

Figure S14. Nyquist plots of IMO and IOMO acquired using the overpotential of 110 mV.

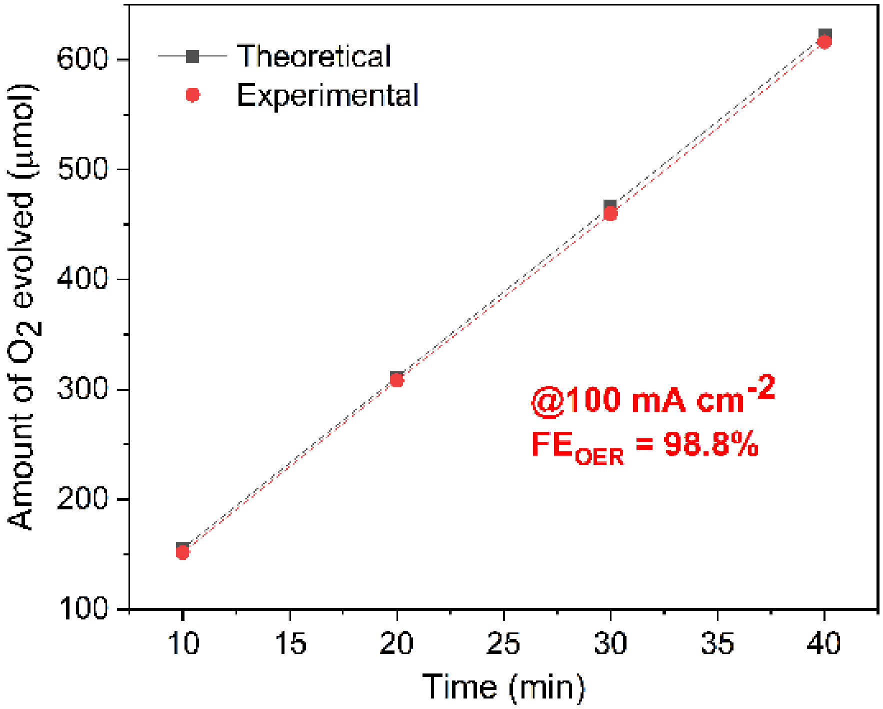

Figure S15. Faradaic efficiency of the IMO electrode showing the amounts of theoretically calculated and experimentally measured O_2 gas over time in 0.5 M H_2SO_4 at current density of 100 mA cm^{-2}.

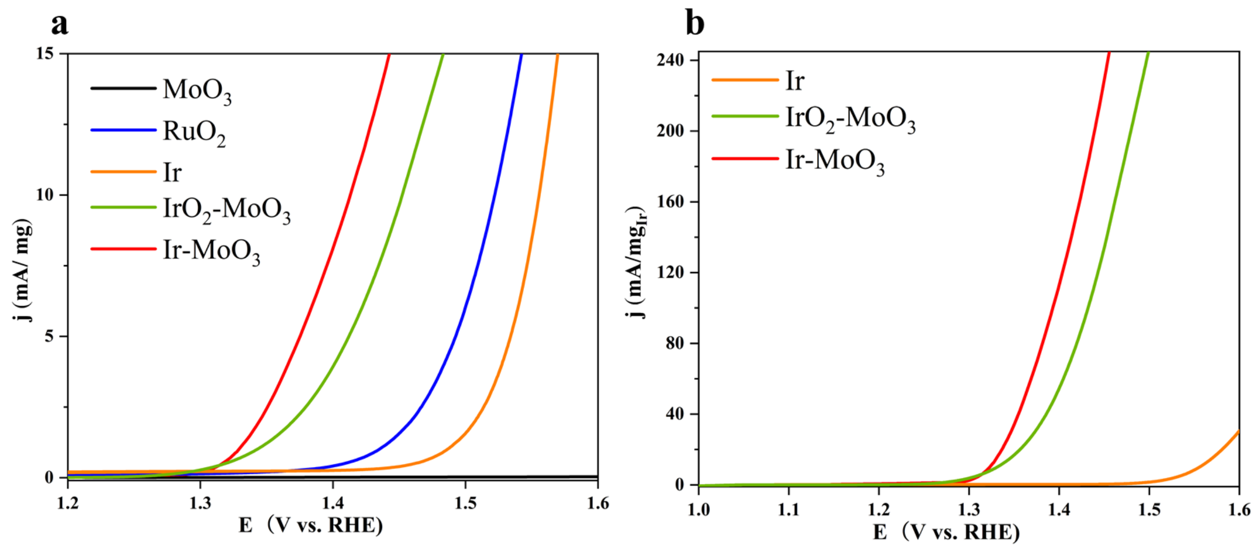

Figure S16. a, The mass activity of as-prepared electrodes normalizing by total loading of catalyst. **b,** The mass activity of as-prepared electrodes normalizing by loading of Ir.

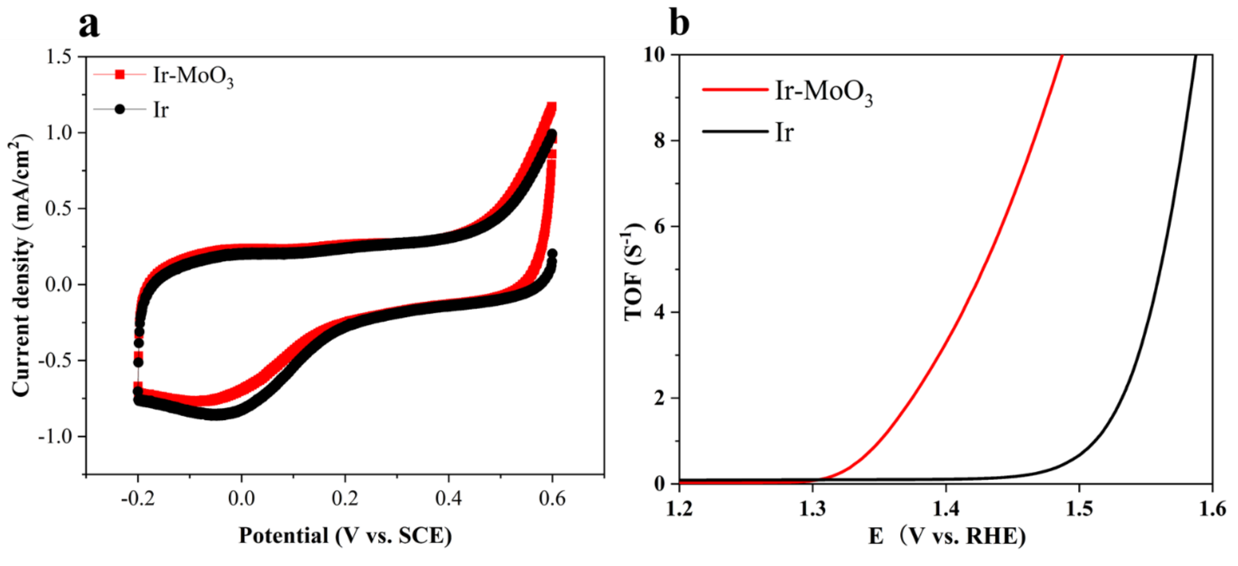

Figure S17. a, the CV curve of IMO and It at a scan rate of 50 mV s^{-1} in PBS solution (pH = 7.4)**. b,** the TOFs of the IMO and Pt in 0.5 M H_2SO_4.

For turnover frequency (TOF) calculation:

The upper limit of the number of active site (N) was first determined by measuring CV curves in 1 mol phosphoric acid buffer (PBS) (pH = 7.4) at a scan rate of 50 mV s^{-1}.[1] The N (mol) and TOF (s^{-1}) were calculated with the following equations:

$$N = \frac{Q}{2F}$$

$$TOF = \frac{I}{2NF}$$

where Q (C) is the number of voltammetric charge, F is the Faraday constant (96485. 3329 C mol^{-1}), and I (A) is the current obtained from the LSV polarization curve.

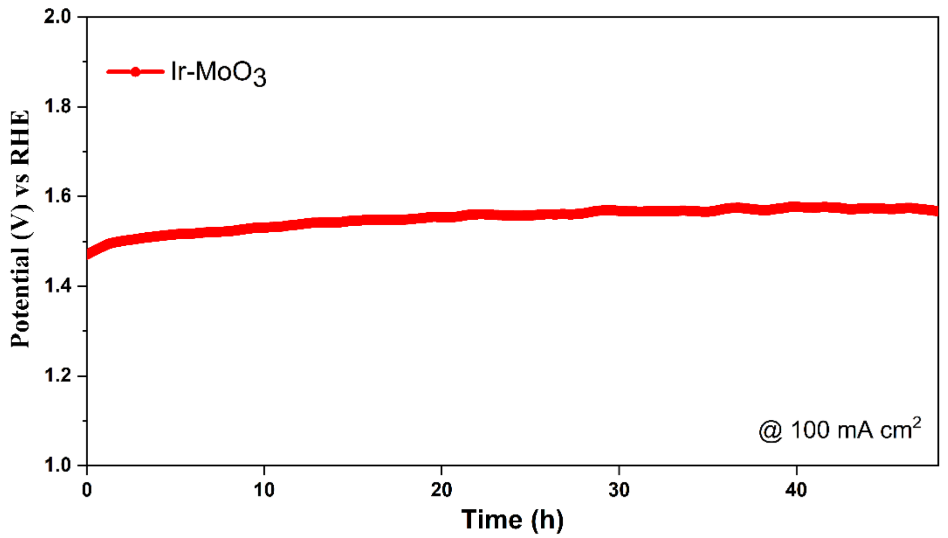

Figure S18. Chronopotentiometric curves of Ir-MoO_3 at a current density of 100 mA cm^2.

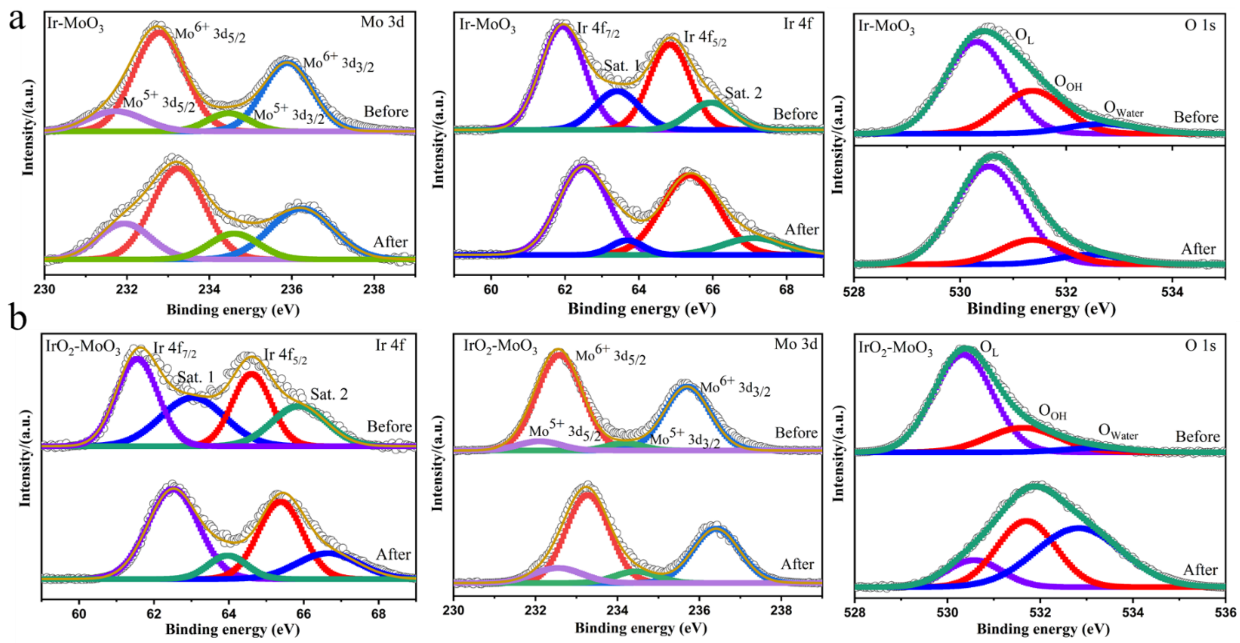

Figure S19. XPS patterns for before and after the OER stability measurement. a, IMO and **b,** IOMO.

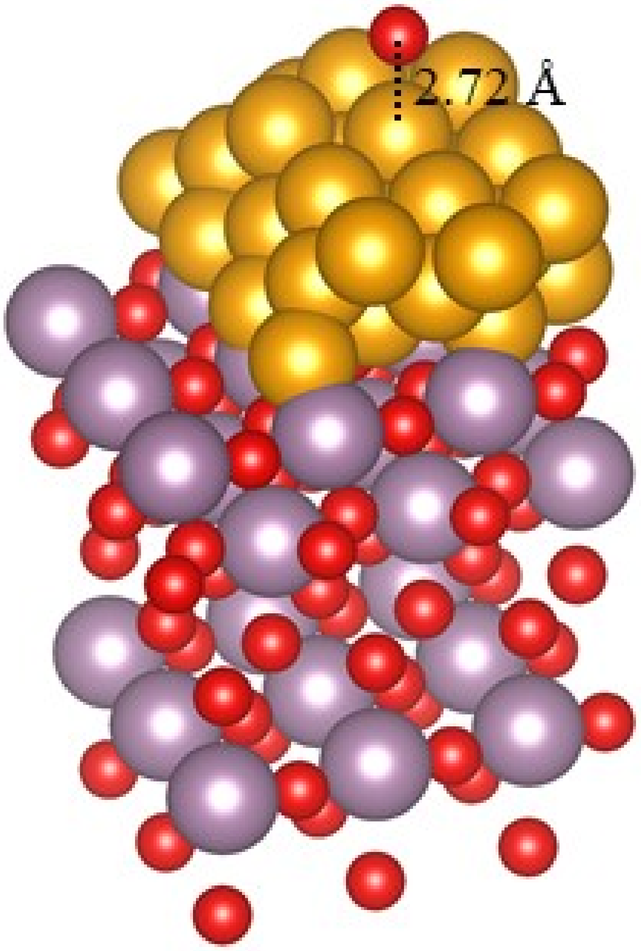

Figure S20. The side view of IMO's relaxed structure for the oxygen adsorbed on the site D. The red, pink, violet, grey, and rosy-brown balls represent the O, H, Co, Ni, and Ru atoms, respectively.

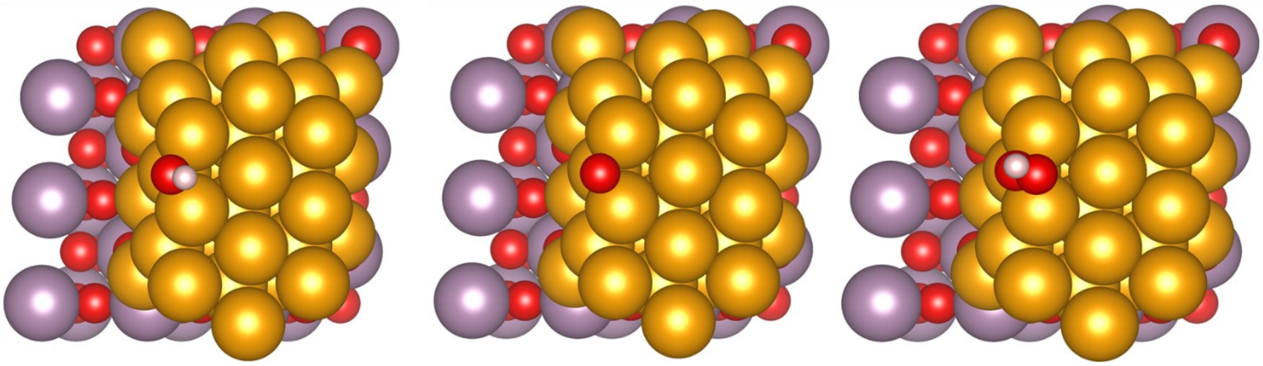

Figure S21.The configuration of the OER process for IMO.

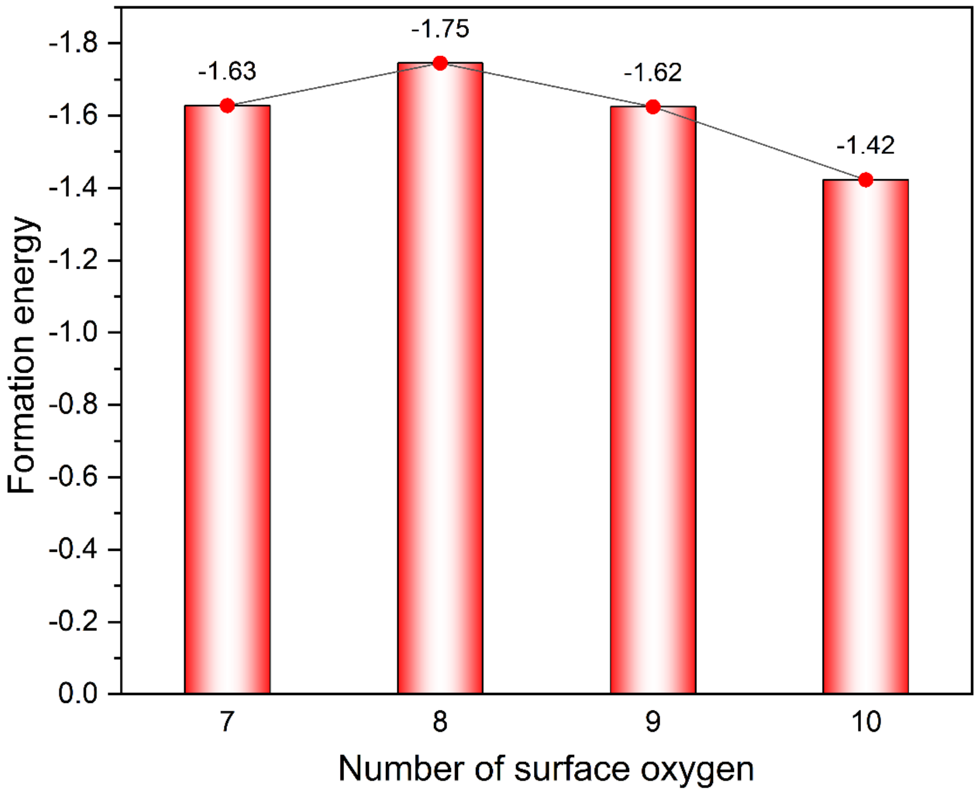

Figure S22. The formation for various amounts of surface oxygen for IMO.

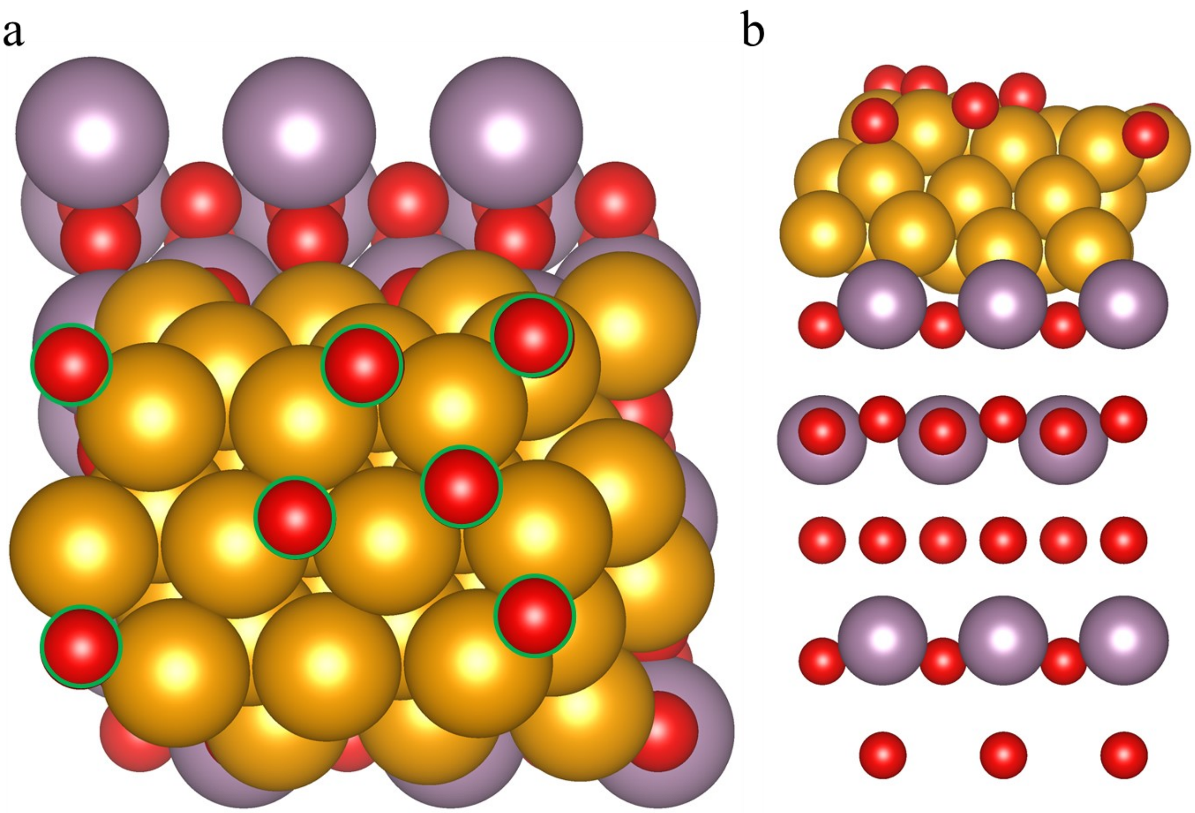

Figure S23. The top and side view of the IMO with 7 surface oxygen atoms.

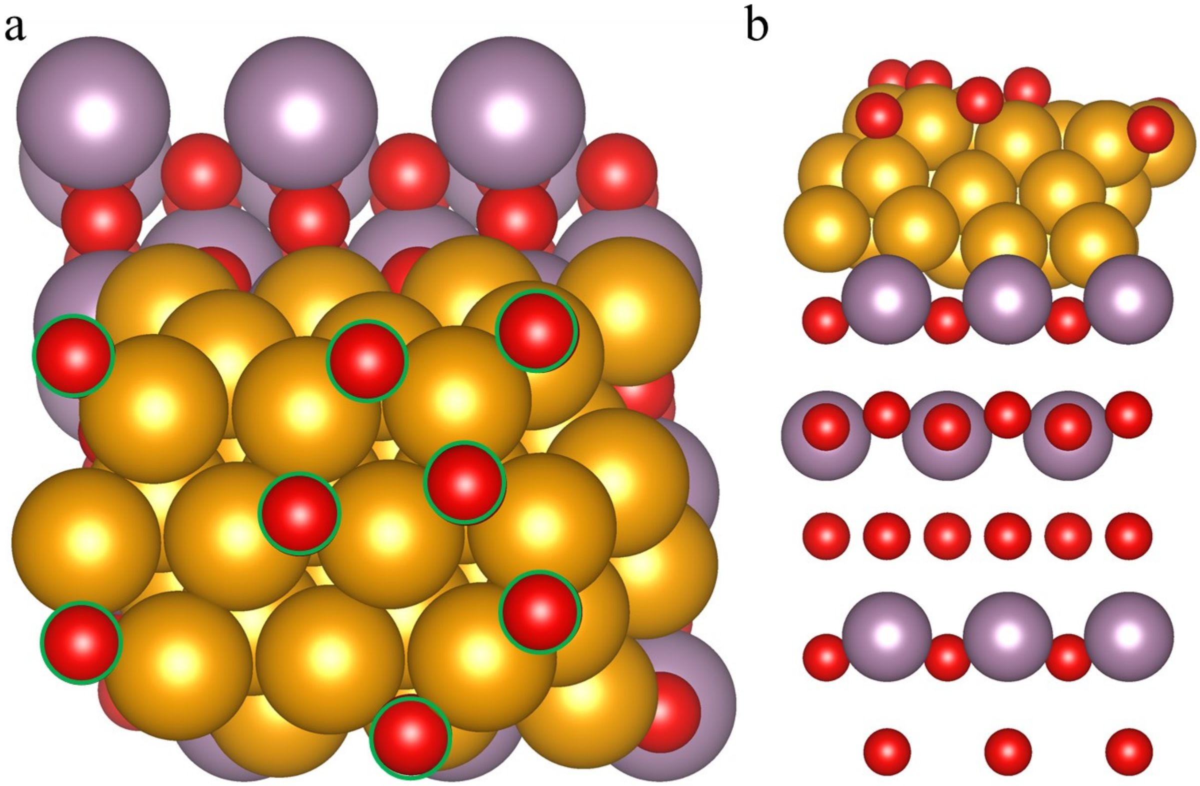

Figure S24. The top and side view of the IMO with 8 surface oxygen atoms.

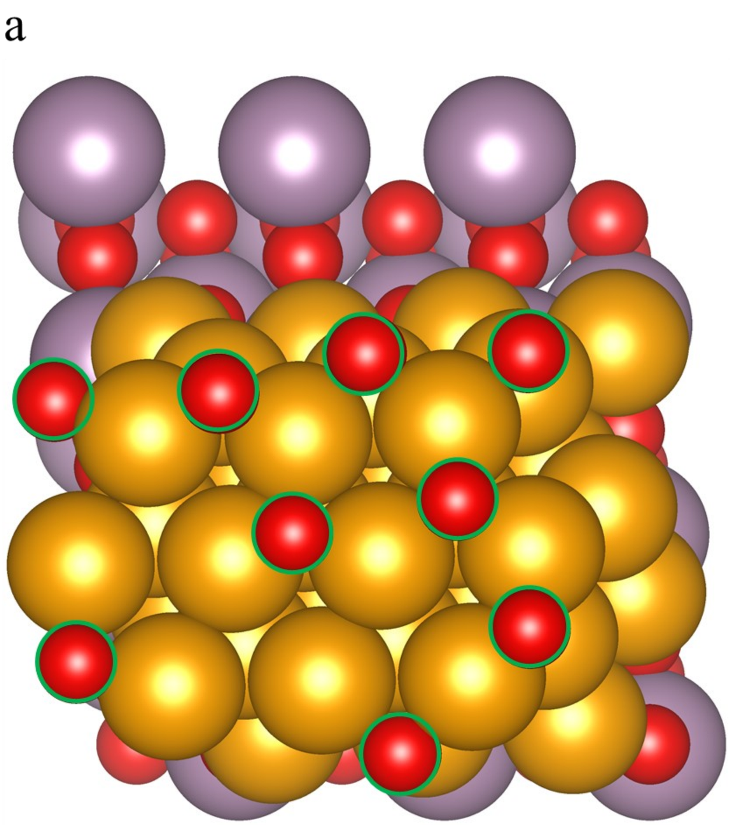

Figure S25. The top and side view of the IMO with 9 surface oxygen atoms.

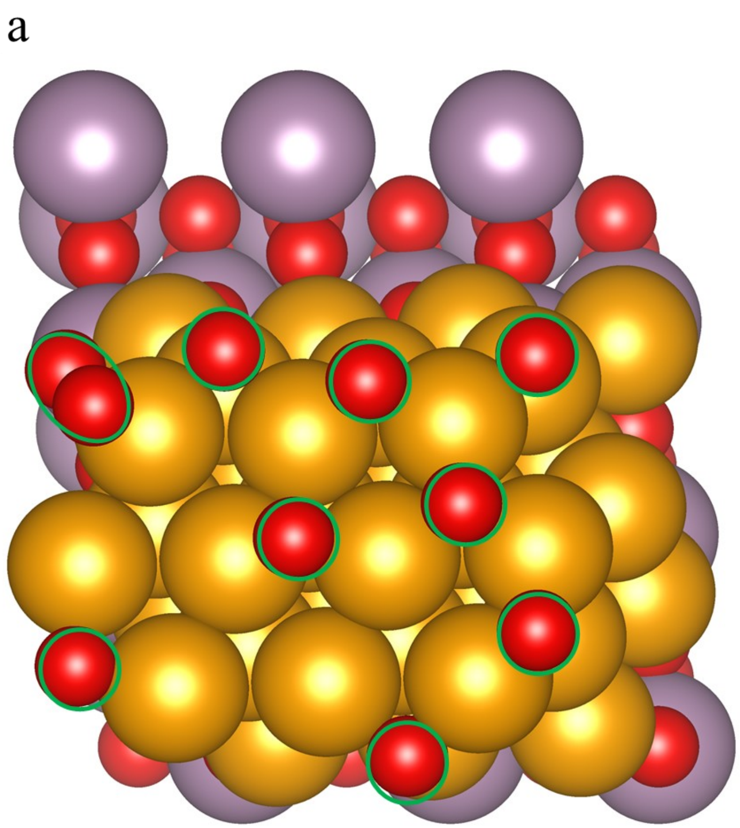

Figure S26. The top and side view of the IMO with 10 surface oxygen atoms.

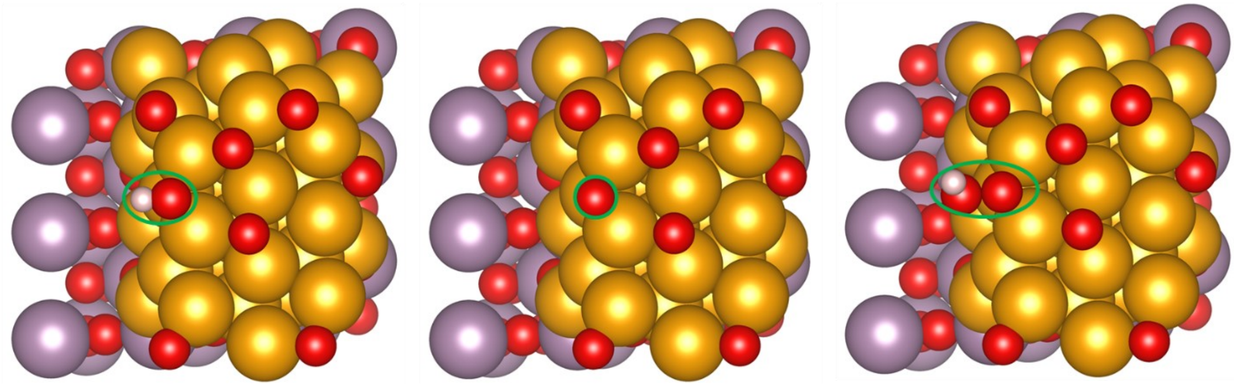

Figure S27.The configuration of the OER process for IMO (O-7).

Table S1. Parameter for deconvolution of XPS spectra.

Material	Peak	Position (eV)	Shape (eV)	FWHM* (eV)	Peak Area
IMO	Ir $4f_{7/2}$	61.93	Gaussian	1.4	2239.5
	Ir $4f_{5/2}$	64.84	Gaussian	1.3	1710.3
	O 1s	530.31	Gaussian	1.4	11418.9
	O 1s	531.36	Gaussian	1.4	5334.9
	O 1s	532.68	Gaussian	1.6	1379.1
	Mo^{5+} $3d_{5/2}$	231.72	Gaussian	1.6	2860.9
	Mo^{6+} $3d_{5/2}$	232.79	Gaussian	1.46	12835.1
	Mo^{5+} $3d_{3/2}$	234.46	Gaussian	1.2	1514.3
	Mo^{6+} $3d_{3/2}$	235.89	Gaussian	1.4	8539.4
IOMO	Ir $4f_{7/2}$	61.55	Gaussian	1.3	1181.2
	Ir $4f_{5/2}$	64.53	Gaussian	1.22	917.7
	O 1s	530.33	Gaussian	1.5	10722.1
	O 1s	531.36	Gaussian	1.6	3366.9
	O 1s	532.66	Gaussian	1.4	739.1
	Mo^{5+} $3d_{5/2}$	232.10	Gaussian	1.2	805.8
	Mo^{6+} $3d_{5/2}$	232.59	Gaussian	1.3	9068.9
	Mo^{5+} $3d_{3/2}$	234.18	Gaussian	1.04	543.97
	Mo^{6+} $3d_{3/2}$	235.70	Gaussian	1.3	6078.51
Ir	Ir $4f_{7/2}$	60.93	Gaussian	1.2	3360.0
	Ir $4f_{5/2}$	63.83	Gaussian	1.23	2583.7
$IrCl_3$	Ir $4f_{7/2}$	61.68	Gaussian	1.8	967.8
	Ir $4f_{5/2}$	64.67	Gaussian	1.7	727.1
IrO_2	Ir $4f_{7/2}$	61.63	Gaussian	1.34	1607.1
	Ir $4f_{5/2}$	64.56	Gaussian	1.4	1204.2
	O 1s	529.83	Gaussian	1.4	1385.1
	O 1s	531.18	Gaussian	1.8	1117.2
	O 1s	533.05	Gaussian	1.8	464.95

***FWHM: Full with at half maximum.**

Table S2. EXAFS fitting parameters various samples

Samples	Path	Coordination number	Radial distance	Debye-Waller factor (σ^2)
**IMO-Ir metal**	Ir-Ir1	12	2.77(2)	0.0037(2)
	Ir-Ir2	6	3.85(3)	0.0042(11)
	Ir-Ir3	24	4.71(4)	0.0057(6)
	Ir-Ir4	12	5.44(4)	0.0047(8)
	Ir-Ir1-Ir4	48	5.44(4)	0.0047(8)
**IOMO-IrO_2**	Ir-O1	7	1.95(6)	0.0025(3)
	Ir-Ir2	2	3.12(10)	0.0026(14)
	Ir-O1	4	3.53(6)	0.0025(3)
	Ir-Ir2	8	3.55(4)	0.0044(8)
**IMO-MoO_3**	Mo-O1	1	1.65(8)	0.0012(8)
	Mo-O2	1	1.72(8)	0.0012(8)
	Mo-O3(Ir)	2	1.93(13)	0.0032(10)
	Mo-O4(Ir)	1	2.28(2)	0.005(2)
	Mo-O5	1	2.35(2)	0.005(2)
	Mo-Mo1	2	3.38(7)	0.0007(8)
	Mo-O6	4	3.35(8)	0.0012(8)
	Mo-Mo2	2	3.78(1)	0.0007(7)
	Mo-Mo3	2	4.02(1)	0.0007(7)
**IOMO-MoO_3**	Mo-O1	1	1.66(8)	0.0014(8)
	Mo-O2	1	1.72(8)	0.0014(8)
	Mo-O3	2	1.93(13)	0.0035(15)
	Mo-O4	1	2.28(3)	0.004(2)
	Mo-O5	1	2.35(3)	0.004(2)
	Mo-Mo1	2	3.37(9)	0.0012(7)
	Mo-O6	4	3.35(8)	0.0014(8)
	Mo-Mo2	2	3.77(9)	0.0015(12)
	Mo-Mo3	2	4.00(1)	0.0015(12)

Radial distance: interatomic distance (the bond length between the central atom and surrounding coordination atoms). $\boldsymbol{\sigma^2}$: Debye-Waller factor.

Table S3. Faradic efficiency for OER (FE_{OER})

**Time (min)**	**O_2 Volume (mL)**	**Experimental O_2 µmol**	**Q**	**Theoretical O_2 (µmol)(**	**FE_{OER}**
10	3.4	151.786	60.008	155.464	98.7
20	6.8	308.036	120.016	310.969	99.01
30	10.3	459.821	180.023	466.392	98.6
40	13.8	616.071	240.031	621.856	99.01

For Faradic efficiency measurement:

The Faradic efficiency was calculated using the equation.

$$\text{Faradic efficiency} = \frac{\text{experimental µmol of } O_2}{\text{Theoretical µmol of } O_2} \times 100$$

The theoretical amount of O_2 gas was calculated from Faraday's law:

$$n = \frac{I \times t}{z \times F}$$

$$n = \frac{Q}{z \times F}$$

where n is the number of mols, I is the current in ampere, t is the time in seconds, z is the transfer of electrons (for O_2 z = 4), and F is the Faraday constant (96485. 3329 C mol^{-1}). Q is charge.

Table S4. Parameter for deconvolution of XPS spectra before/after the OER reaction for IMO and IOMO.

Material	Peak	Position (eV)	Shape (eV)	FWHM* (eV)	Peak Area
IMO	Ir $4f_{7/2}$	61.93	Gaussian	1.4	2239.5
	Ir $4f_{5/2}$	64.84	Gaussian	1.3	1710.3
	O 1s	530.31	Gaussian	1.4	11418.9
	O 1s	531.36	Gaussian	1.4	5334.9
	O 1s	532.68	Gaussian	1.6	1379.1
	Mo^{5+} $3d_{5/2}$	231.72	Gaussian	1.6	2860.9
	Mo^{6+} $3d_{5/2}$	232.79	Gaussian	1.46	12835.1
	Mo^{5+} $3d_{3/2}$	234.46	Gaussian	1.2	1514.3
	Mo^{6+} $3d_{3/2}$	235.89	Gaussian	1.4	8539.4
Used IMO	Ir $4f_{7/2}$	62.64	Gaussian	1.7	358.15
	Ir $4f_{5/2}$	65.64	Gaussian	1.74	268.82
	O 1s	530.54	Gaussian	1.5	11838.4
	O 1s	531.36	Gaussian	1.4	2753.67
	O 1s	532.46	Gaussian	1.45	1139.73
	Mo^{5+} $3d_{5/2}$	231.94	Gaussian	1.5	741.41
	Mo^{6+} $3d_{5/2}$	233.24	Gaussian	1.5	1879.54
	Mo^{5+} $3d_{3/2}$	234.60	Gaussian	1.4	498.31
	Mo^{6+} $3d_{3/2}$	236.23	Gaussian	1.8	1262.28
IOMO	Ir $4f_{7/2}$	61.55	Gaussian	1.3	1181.2
	Ir $4f_{5/2}$	64.53	Gaussian	1.22	917.7
	O 1s	530.33	Gaussian	1.5	10722.1
	O 1s	531.36	Gaussian	1.6	3366.9
	O 1s	532.66	Gaussian	1.4	739.1
	Mo^{5+} $3d_{5/2}$	232.10	Gaussian	1.2	805.8
	Mo^{6+} $3d_{5/2}$	232.59	Gaussian	1.3	9068.9
	Mo^{5+} $3d_{3/2}$	234.18	Gaussian	1.04	543.97

	Mo^{6+} $3d_{3/2}$	235.70	Gaussian	1.3	6078.51
Used IOMO	Ir $4f_{7/2}$	62.51	Gaussian	1.6	1499.94
	Ir $4f_{5/2}$	65.38	Gaussian	1.4	1127.94
	O 1s	530.57	Gaussian	1.4	1829.78
	O 1s	531.70	Gaussian	1.6	4796.47
	O 1s	532.83	Gaussian	1.4	6277
	Mo^{5+} $3d_{5/2}$	232.54	Gaussian	1.4	424.23
	Mo^{6+} $3d_{5/2}$	233.27	Gaussian	1.24	2217.66
	Mo^{5+} $3d_{3/2}$	234.45	Gaussian	1.2	280.78
	Mo^{6+} $3d_{3/2}$	236.41	Gaussian	1.34	1476.57

References:

1. Yu F*, et al.* High-performance bifunctional porous non-noble metal phosphide catalyst for overall water splitting. *Nature Communications* **9**, 2551 (2018).
